# Donor Heart Utilization following Cardiopulmonary Arrest and Resuscitation: Influence of Donor Characteristics and Wait Times in Transplant Regions

**DOI:** 10.1155/2014/519401

**Published:** 2014-07-08

**Authors:** Mohammed Quader, Luke Wolfe, Gundars Katlaps, Vigneshwar Kasirajan

**Affiliations:** Department of Cardiothoracic Surgery, Virginia Commonwealth University, Richmond, VA 23298, USA

## Abstract

*Background*. Procurement of hearts from cardiopulmonary arrest and resuscitated (CPR) donors for transplantation is suboptimal. We studied the influences of donor factors and regional wait times on CPR donor heart utilization. *Methods*. From UNOS database (1998 to 2012), we identified 44,744 heart donors, of which 4,964 (11%) received CPR. Based on procurement of heart for transplantation, CPR donors were divided into hearts procured (HP) and hearts not procured (HNP) groups. Logistic regression analysis was used to identify predictors of heart procurement. *Results*. Of the 4,964 CPR donors, 1,427 (28.8%) were in the HP group. Donor characteristics that favored heart procurement include younger age (25.5 ± 15 yrs versus 39 ± 18 yrs, *P* ≤ 0.0001), male gender (34% versus 23%, *P* ≤ 0.0001), shorter CPR duration (<15 min versus >30 min, *P* ≤ 0.0001), and head trauma (60% versus 15%). Among the 11 UNOS regions, the highest procurement was in Region 1 (37%) and the lowest in Region 3 (24%). Regional transplant volumes and median waiting times did not influence heart procurement rates. *Conclusions*. Only 28.8% of CPR donor hearts were procured for transplantation. Factors favoring heart procurement include younger age, male gender, short CPR duration, and traumatic head injury. Heart procurement varied by region but not by transplant volumes or wait times.

## 1. Introduction

**Figure 1:**
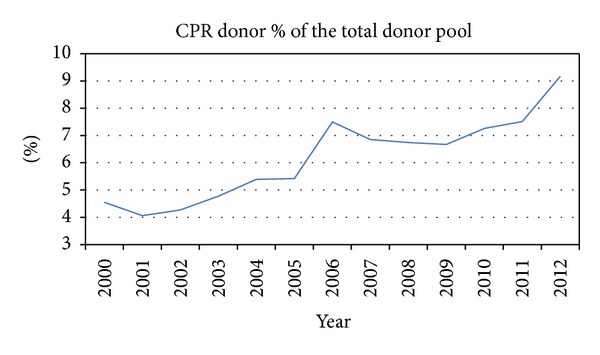
CPR donor percentage of the total heart donors.

For patients with advanced heart failure awaiting heart transplantation (HTx), donor heart supply remains a limiting factor in offering the ultimate treatment option. Efforts to optimize management of potential heart donors have led to increased utilization of donor hearts [1], yet this increase falls far short of the existing demands on organs for transplantation [2]. New avenues that would increase available donor hearts have been explored, including donation after cardiac death [3], ex vivo organ resuscitation [4], and, importantly, extended donor selection criteria [5]. Of these extended criteria, cardiopulmonary arrest and resuscitated (CPR) organ donors have significantly increased the potential organ donor pool. In the past decade alone, there has been a 90% increase in the number of organ donors who were successfully resuscitated after cardiopulmonary arrest [2] (Figure 1). We previously reported that the clinical outcomes of heart transplantation from CPR donors are similar to the outcomes from non-CPR donors [6]. This finding was also noted in other solid organ transplantation studies [7, 8].

Despite these encouraging reports, utilization of CPR donor hearts has been less than 30% [2]. The reasons are multifactorial, including concerns regarding warm ischemic damage to organs sustained during cardiopulmonary arrest [9], nonuniform donor selection criteria among transplantation centers, acuity of illness of the organ recipients, and, perhaps, the overall transplant volumes and experiences. Most published studies on this subject have focused on the recipient outcomes with donor variables studied primarily to predict posttransplantation outcomes [10, 11]. Our study principally focuses on the donor variables, with the aim of identifying CPR donor characteristics predictive of heart procurement for transplantation. We also sought to study the influences of 11 UNOS regional heart transplantation volumes and regional wait times on CPR donor heart utilization.

## 2. Material and Methods

**Figure 2:**
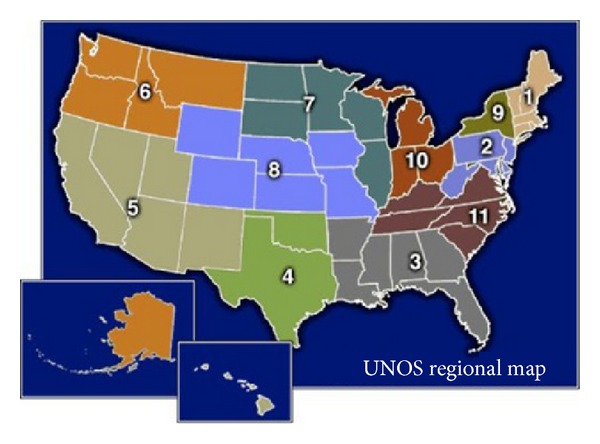
UNOS 11 heart transplantation regions in USA.

We examined the UNOS heart donor data between 1998 and 2012 and identified 44,744 consented heart donors, of which 4,964 (11%) were from CPR donors. This comprised our study group, which was further divided into two groups based on heart procured (HP) or heart not procured (HNP) for transplantation. Besides donor demographics, data on comorbid conditions, social history, causes of death, and duration of CPR were collected in a deidentified fashion for analysis (Table 1). Data on 11 UNOS transplantation regions (Figure 2) were gathered and analyzed for transplantation volumes, median 1A status wait times for heart transplantation, and percent CPR donor heart utilization (Table 2).

Numeric data were analyzed and reported as mean, median, and standard deviations. A *P* value of less than 0.05 is considered statistically significant. Continuous variables were analyzed with Student's *t*-test; categorical variables were analyzed with the chi-square test. A logistic regression analysis of 11 donor variables was performed to identify predictors of heart procurement for transplantation from the CPR donor pool. The results of this regression analysis are reported as odds ratios with confidence limits.

## 3. Results

During the study period of 1998 to 2012, a total of 44,744 consented heart donors were identified in the UNOS database. Of which, 4,964 (11%) donors sustained cardiopulmonary arrest and were resuscitated to spontaneous rhythm and circulation. Only 1,427 (28.8%) of these CPR donor hearts were procured for transplantation; these donors comprised the hearts procured (HP) group.

When compared to the hearts not procured group, the hearts procured group were relatively younger (25 ± 15 years versus 39 ± 18 years, *P* ≤ 0.0001) and a high proportion of them were male gender (34% M, versus 23% F, *P* ≤ 0.0001). Cause of death influenced the percent utilization of CPR donor hearts: those with head trauma leading to brain death were selected more often for heart procurement compared to those with intracerebral bleed (60% and 15%, resp., *P* ≤ 0.0001). Similarly, mechanism of death played a role in the selection of CPR donors for heart procurement: those with gunshot wounds (44%), asphyxiation (40%), or blunt trauma (39%) were more often selected for heart procurement compared to those with stroke (15%). CPR donors with a history of hypertension were selected less often for heart procurement, as only 12% of the hearts procured group had a history of hypertension, compared to 35% in the hearts not procured group (*P* ≤ 0.0001). Ethnicity and the social history of the CPR donor were also factored into the heart procurement for transplantation (Table 1). Notably, a higher percentage of donors with Hispanic ethnicity (37%) were accepted for heart procurement compared to only 24% for Asian ethnicity (*P* ≤ 0.0001). We noticed that there was a higher prevalence of head trauma (35%), male gender (62%), and blood group O (60%) in Hispanic ethnicity heart donors compared to other heart donors. Donors with a history of tobacco abuse (17%) and heavy alcohol consumption (22%) were less often selected for heart procurement. Duration of CPR also contributed significantly to the selection of donor heart for transplantation. Those donors with a CPR duration of less than 15 min were selected more often for heart procurement than those with a CPR duration of greater than 30 min (31% and 25%, resp., *P* ≤ 0.0001). Left ventricle ejection fraction (EF) was near normal in heart procured group compared to heart not procured group, 61.4 ± 9.0 versus 48.2 ± 17.3, *P* < 0.0001.

Data on regional heart transplantation volumes, wait times on the transplantation list, and CPR donor heart utilization were also studied (Table 2). Heart transplantation volumes, measured as the mean number of transplantations performed per year, varied between the 11 UNOS regions, from 64 in Region 6 to 337 in Region 5. Median wait times on status 1A listing also varied significantly between the regions and did not correlate with transplantation volumes. The longest median wait time was noted in Region 7 (90 days) while the shortest was in Region 5 (35 days).

CPR donor heart utilization varied significantly between the eleven regions, with a low of 24% in Region 3 to a high of 37% in Region 1 (*P* ≤ 0.0001). We also evaluated the CPR donor utilization by population per heart transplantation performed in the 11 regions. Region 6 had the highest population per heart transplant (242,517/HTx), while the lowest was in Region 2 (110,026/HTx). CPR donor utilizations in Regions 6 and 2 were 26% and 29%, not statistically significant.

Logistic regression analysis using heart procurement for transplantation from CPR donors as an outcome variable was studied factoring in eleven donor variables, including regional transplant volumes, and wait times on transplantation list (Table 3). CPR donor characteristics associated with heart procurement for transplantation by logistic regression analysis include younger donor age, male gender, shorter CPR duration, head trauma as a cause of death versus cerebrovascular accident, Hispanic ethnicity, blood group O, and location in Region 1. Factors that were negatively associated with heart procurement for transplantation were need for multiple inotropes at the time of organ procurement, history of substance abuse, nontraumatic injuries leading to brain death, low EF, and location in Region 3 (Table 3).

## 4. Comment

**Figure 3:**
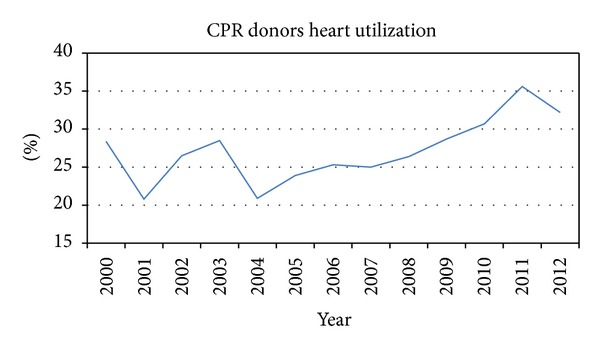
CPR donor heart procurement for transplantation between 2000 and 2012.

Since the limiting factor in offering heart transplantation to patients with advanced heart failure is a suitable heart donor, we embarked on studying a group of underutilized potential heart donors: those who sustained cardiopulmonary arrest and were resuscitated. Our study demonstrates that CPR donors that are younger, are male, incurred head trauma, received shorter duration CPR, and did not smoke or abuse alcohol are selected more often for heart procurement. We did not find a correlation between CPR donor heart utilization and regional transplantation volumes or wait times. This information is unique to our study and has not been studied and reported before. We noted a small but encouraging difference in utilization of CPR donor hearts over time, from 28.4% in 2000 to 32.2% in 2012 (Figure 3).

With the implementation of onsite CPR protocols, including both the advanced training of emergency medical response personnel and the increased availability of automated electrical defibrillators to the lay public, an increasing number of cardiopulmonary arrest victims are receiving CPR onsite and are being brought to the hospitals for continued resuscitation [12]. Unfortunately, a large percentage of these patients, as high as 70% arriving to hospitals after an onsite resuscitation with return of spontaneous circulation, are sustaining severe permanent brain damage [13, 14] and are then evaluated for organ donation. Over the past decade, the number of CPR donors have increased from 4.8% of the total donor pool in the year 2000 to 9.1% in the year 2012 (Figure 1); a 90% increase over time [2]. However, concerns remain regarding the optimal posttransplantation outcomes from CPR donors [9, 15], despite multiple studies demonstrating noninferior outcomes after heart transplantation from such donors [6, 16]. Presently, only one-third of the potential CPR donor hearts are procured for heart transplantation [2]. However, there has been an upward trend in accepting hearts from these donors (Figure 3).

Donor factors associated with suboptimal posttransplantation outcomes have been studied extensively. Older donor age [5, 10], hypertension, low EF [10, 17], diabetes [11, 17], excess alcohol intake [18], and cigarette smoking [19] are among the well-established risk factors. In our study, presence of these factors among the CPR donors was associated with less procurement rates of hearts for transplantation, correlating with the published literature.

Donor gender significantly influences the heart utilization rate. In our study, we noted male CPR donor heart procurement of 34% as compared to female donor heart procurement of only 23%; this, too, is consistent with most of the other studies. In a large study of over 1800 organ donors, 72% of male donor organs were utilized for transplantation as compared to 28% of female donor organs [10]. The male donor gender preference is primarily influenced by the larger male donor body size that matches well for most male and for all female recipients. Additionally, heart transplantation from female donors has been associated with less than optimal posttransplantation outcomes, both early and late, especially if the recipients were male [20].

An organ donor's cause of death has been known to influence posttransplantation outcomes. In particular, death resulting from cerebrovascular accidents and intracranial bleeding is associated with poor posttransplantation outcomes [10, 21]. This explains the low percentage utilization of hearts from the CPR donors with strokes in our study. The likely explanation for this observation is that perhaps those donors who develop massive strokes tend to be relatively older and carry significant cardiovascular risk factors, such as hypertension and diabetes. Both of these factors are associated with inferior posttransplantation outcomes.

Studies on mechanism of injury leading to brain death show that this also influences donor organ utilization. Trauma victims constitute 55% to 74% of organ donors [22]. In a study by Kutschmann et al., besides older donor age, nontraumatic causes of death were associated with decreased recipient postheart transplant survival with an odds ratio of 1.48 [23]. Similarly, another study demonstrated that traumatic head injury leading to brain death is associated with higher donor organ utilization: 61% versus 22% for donors without head injury as a cause of brain death [10]. Our study's findings also suggest similar CPR donor heart procurement from both traumatic injury donors and donors with head trauma leading to brain death.

Organ donation rates differ among ethnic groups and are influenced by cultural beliefs and practices. In our study, we observed different rates of CPR donor heart procurement for transplantation by ethnicity. Donor heart procurement for transplantation from Hispanics, African Americans, Caucasians, and Asian ethnic groups was 37%, 33%, 27%, and 24%, respectively. This disparity in donor heart selection was also noted in another study where proportionately more Hispanic (30%) and fewer Asian donors (5%) were selected for heart procurement for transplantation following brain death compared to other ethnicities studied [10]. In our study, higher prevalence of head trauma (35%), male gender (62%), and blood group O (60%) was noted in Hispanic ethnicity heart donors compared to other heart donors; it is possible that these attributes in isolation or in combination have contributed to higher heart procurement from Hispanic ethnicity heart donors.

The most common blood group among US population is O (44%) followed by A (42%), B (10%), and AB (4%). Since donors and recipients are matched primarily by blood groups, it is expected that donors with O blood group (so-called universal donors) would be utilized most frequently. Heart procurement for transplantation among CPR donors in our study is consistent with this expectation.

Transplantation volumes and duration of wait times on the transplantation list vary at different centers and depend on the prevalence of heart failure within the patient population, the number of competing programs in the region, and, most importantly, the surrounding population. Posttransplantation outcomes have been linked to a center's transplantation volume, with low volume centers showing inferior survival [24]. In a study by Hosenpud et al., the critical heart transplantation center volume was nine per year; below this, survival outcomes were considered poor [25]. In another study by Kilic et al., institutional factors were also identified as contributing to inferior posttransplantation outcomes [26]. There, the authors suggest that the “heart transplantation center's volume” should not be the sole indicator of center's quality in heart transplantation. When donor risk factors were taken into account and posttransplantation outcomes were compared to transplant center volumes, it was clear that the low-volume centers did poorly with high-risk donors [26]. The difference in posttransplantation outcome persisted even when both the recipient risk factors and the institutional factors were taken into account. When stratified by the recipient risk scores, the posttransplantation outcomes appear profoundly worse in transplantation of extended criteria donors to high-risk recipients. The authors concluded that consolidating the use of extended criteria donors to higher volume centers might be prudent in improving postheart transplantation outcomes in high-risk recipients. The findings of the Kilic et al. study should be interpreted with caution, as the donor variables studied were few and transplant volumes defined as “low” were below 14 heart transplants per year. To follow this recommendation, it would prohibit greater than 77% of heart transplantation centers in the US from using high-risk donors [19]. In the same study, extended criteria donor utilization varied by the UNOS regions, with Region 8 utilizing 11.4% of marginal donors compared to 24.7% in Region 9 [26]. In our study, the highest percentage utilization of CPR donor hearts was in Region 1 (37%) and the lowest was in Region 3 (24%). We believe the difference in our results stems from different definitions of donor heart utilization: high-risk versus CPR donors. Time on 1A status varied significantly among the 11 regions, from a median of 35 days in Region 5 to 90 days in Region 7. It is possible to assume that the regions with longer wait times would likely accept extended criteria donors more often, including those who received CPR. In our study, however, longer wait times in regions did not correlate with increased CPR donor heart utilization. Similarly, our findings on the CPR donor heart utilization by population per heart transplantation performed in the 11 regions did not support any correlation between population dense regions and CPR donor heart utilization.

## 5. Study Limitations

Limitations inherent to retrospective data analysis are applicable to our study. The primary data source for our study is a composite UNOS database from more than 150 US transplantation centers. Completeness of all data fields is wanted and is not always available in the database. However, its merit lies in the fact that large, pooled data from different transplant centers eliminate biases associated with individual center practices. Thus, certain generalizations can be made of the results with regard to practicality of its usage and reproducibility. Since our focus was on the characteristics of CPR donors only, this limited the study population to 11% of the total donor pool, thus eliminating a majority of donors from our analysis. Since CPR donors comprise a potential group of underutilized heart donors, we felt it is important that we focus on this small but important group of donor hearts. We used the large UNOS database to help define donor characteristics that were associated with heart procurement for transplantation with the hope that in future at least CPR donor hearts with similar characteristics would be utilized more frequently for transplantations. Accepting a heart from an extended criteria donor also depends on the medical condition and acuity of illness of the recipient. The present study has not taken the recipient factors into consideration when defining CPR donor heart utilization. In our previous study of CPR heart donors, we noticed that more CPR donors were matched to recipients with high acuity of illness at transplantation, such as those with status 1A listing, those that were admitted to the hospital at transplantation, and those that were supported in an intensive care unit on life-sustaining support [6]. Despite the higher acuity of illness in the recipients, the use of selected CPR donor hearts did not negatively influence the intermediate or long-term outcomes of heart transplantation. Transplantation center practices do change over time with changes in leadership, philosophy, and patient population. We did not study the temporal changes in CPR donor heart utilization over time for each transplant region, or for each transplant center. With over 150 transplantation centers in 11 UNOS regions, we feel that 15 years of temporal data would be both cluttered in presentation and not very meaningful to the reader.

## 6. Conclusions

CPR donors are an expanding group of potential, yet presently underutilized, heart donors. With improvements in onsite resuscitative measures, more patients are regaining spontaneous circulation after CPR. However, if irreversible neurologic damage is sustained, these patients become eligible for organ donation. Though the clinical outcomes from accepting hearts from these donors are comparable, the overall utilization of these donor hearts is only 28.8%. Donor characteristics such as younger age, male gender, absence of hypertension, smoking history, traumatic head injury, and shorter CPR duration are associated with higher heart procurement rates for transplantation. Regional factors do play a role in donor heart selection, but it does not seem to be associated with either recipient wait times or regional transplantation volumes. It is our hope that in the future CPR donor hearts with attributes associated with successful heart procurement for transplantation will not be passed up and that serious efforts will be made to accept these hearts, thus helping patients waiting desperately for transplantation.

## Figures and Tables

**Table 1:** CPR donor characteristics.

Donor variable	Heart procured for transplantation		*P* value
	Yes	No	
	*N* = 1,427 (28.8%)	*N* = 3,537 (67.2%)	
Age in years	25.5 ± 15 yrs	39 ± 18 yrs	<0.0001

Gender			<0.0001
Male	34	66	
Female	23	77	

Duration of CPR			<0.0001
<15 min	31	69	
15–30 min	29	71	
>30 min	25	75	

Cause of Death			<0.0001
Head trauma	60	40	
Anoxia	29	71	
Cerebrovascular/ stroke	15	85	

Ethnicity			<0.0001
Hispanic	37	63	
Black	33	67	
White	27	73	
Asian	24	76	

Donor ABO			<0.0001
O	32	68	
A	27	73	
B	24	76	
AB	15	85	

Hypertension			<0.0001
Yes	12	88	
No	35	65	

Social history			
Cigarette use			<0.0001
Yes	17	83	
No	34	66	
Heavy ETOH use			0.0001
Yes	22	78	
No	30	70	

UNOS region			<0.0075
1	37	63	
3	24	76	

3 or more inotropic agents at the time of incision			<0.0001
Yes	12	88	
No	30	70	

LV ejection fraction %	61.4 ± 9.0	48.2 ± 17.3	<0.0001

CPR: cardiopulmonary resuscitation; ABO: blood groups; ETOH: alcohol; UNOS: United Network of Organ Donation; LV EF: left ventricle ejection fraction.

**Table 2:** Heart transplantation volumes, median wait times, and CPR donor heart procured for transplantation by 11 UNOS regions.

Region	Total population	Population/HTx	Mean HTx volume/yr	Median 1A wait time in days	CPR donor utilization-%
1	13,936,692	158,371	88	59.6	37
2	30,917,426	110,026	281	74.3	29
3	48,262,570	165,851	291	40	24
4	29,874,023	140915	212	47.6	27
5	52,294,441	155,176	337	34.6	32
6	15,521,147	242,517	64	72.6	26
7	25,513,744	125,683	203	90.3	30
8	19,601,598	141,018	139	80.3	30
9	20,196,272	133,750	151	58.3	29
10	27,974,919	136,463	205	68.6	28
11	33,498,321	140,160	239	67.6	31

HTx: heart transplantation; CPR: cardiopulmonary resuscitation.

**Table 3:** CPR donor variables that are predictive of successful heart procurement for transplantation by logistic regression analysis.

Donor variable	Odds ratio	95% confidence limits	*P* value
Age in years	1.033	1.028–1.038	<0.0001

Gender			
Female versus male	1.470	1.272–1.699	<0.0001

Comorbidities			
Hypertension: yes versus no	1.981	1.591–2.467	<0.0001

Duration of CPR	1.013	1.008–1.017	<0.0001

Cause of death versus head trauma			
Anoxia	1.118	0.945–1.322	0.2190
Cerebrovascular/ stroke	1.445	1.147–1.820	0.5109

Ethnicity versus Hispanic			
White	1.261	1.012–1.570	0.5255
Black	0.939	0.725–1.215	0.2575
Asian	1.125	0.626–2.020	0.9644

ABO versus O			
A	1.181	1.014–1.376	0.0002
AB	3.278	2.071–5.189	<0.0001
B	1.617	1.287–2.032	0.8244

Social history			
Cigarette use: yes versus no	1.288	1.062–1.563	0.0103
History of cocaine use	1.981	1.591–2.467	0.3330

UNOS region versus 1			
2	1.634	0.990–2.696	0.1654
3	2.983	1.781–4.997	<0.0001
4	2.922	1.625–5.252	0.0047
5	1.835	1.078–3.124	0.9761
6	2.670	1.368–5.211	0.0940
7	1.676	1.006–2.793	0.3316
8	1.658	0.917–2.999	0.5365
9	1.548	0.870–2.754	0.2687
10	1.564	0.940–2.601	0.0910
11	1.762	1.053–2.947	0.6623

LV ejection fraction	0.920	0.912–0.928	<0.0001

3 or more inotropic agents at the time of incision			
Yes versus no	4.859	3.396–6.952	<0.0001

CPR: cardiopulmonary resuscitation; ABO: blood groups; UNOS: United Network of Organ Sharing; LV: left ventricle.
